# Biocontrol-based strategies for improving soil health and managing plant-parasitic nematodes in coffee production

**DOI:** 10.3389/fpls.2023.1196171

**Published:** 2023-06-20

**Authors:** Kanan K. Saikai, Celestine Oduori, Evans Situma, Simon Njoroge, Ruth Murunde, John W. Kimenju, Douglas W. Miano, Solveig Haukeland, Danny Coyne

**Affiliations:** ^1^ International Institute of Tropical Agriculture (IITA), Nairobi, Kenya; ^2^ Agro-Systems Research Group, Wageningen University and Research, Wageningen, Netherlands; ^3^ Department of Plant Science and Crop Protection, The University of Nairobi, Nairobi, Kenya; ^4^ International Centre of Insect Physiology and Ecology (icipe), Nairobi, Kenya; ^5^ Real IPM Co. (Kenya) LTD., Thika, Kenya; ^6^ The Norwegian Institute of Bioeconomy Research (NIBIO), Ås, Norway

**Keywords:** root-knot nematode, *Meloidogyne hapla*, biological control, nematode management, nematode community

## Abstract

Coffee is an important commodity for Kenya, where production is steadily declining, despite a global rise in demand. Of the various constraints affecting production, plant-parasitic nematodes are a significant, but often overlooked, threat. As a perennial crop, treating plantations once infected with nematodes becomes difficult. The current study evaluated the drenching application of two biocontrol agents, *Trichoderma asperellum* and *Purpureocillium lilacinum*, for their nematode control efficacy, as well as their impact on the soil nematode community structure on mature, established coffee trees in Kenya. Seven Arabica coffee field trials were conducted over two years on trees of various ages. All the fields were heavily infested with *Meloidogyne hapla*, the first report of the species on coffee in Kenya. Both fungal biocontrol agents were detected endophytically infecting roots and recovered from soil but not until six months after initial applications. The population densities of *M. hapla* had significantly declined in roots of treated trees 12 months after the initial application, although soil nematode density data were similar across treatments. Based upon the maturity index and the Shannon index, treatment with *T. asperellum* led to improved soil health conditions and enrichment of diversity in the microbial community. Application of *P. lilacinum*, in particular, led to an increased abundance of fungivorous nematodes, especially *Aphelenchus* spp., for which *P. lilacinum* would appear to be a preferred food source. The soils in the trials were all stressed and denuded, however, which likely delayed the impact of such treatments or detection of any differences between treatments using indices, such as the functional metabolic footprint, over the period of study. A longer period of study would therefore likely provide a better indication of treatment benefits. The current study positively demonstrates, however, the potential for using biologically based options for the environmentally and climate-smart management of nematode threats in a sustainable manner on established, mature coffee plantations.

## 1 Introduction

Worldwide, the trade in coffee (*Coffea arabica, C. canephora*) is rising, driven by its increasing consumption as a beverage, including in Africa (International Coffee Organization, 2022). This global increase in demand is largely supported by rapidly expanding plantations in Asia (Krishnan, 2017). So, with increasing demand, why is Africa’s coffee production declining? In Africa, coffee supports the livelihoods of some 10.9 million African farmers, who produce 15.7 million bags per crop year, and which accounts for approximately 13% of global production (International Coffee Organization, 2015). Across East Africa, a general decline in coffee production is attributed to various factors, including loss of land to real estate. But for remaining plantations, pests and diseases rank high among the reasons for production losses (Gitonga and Townsend, 2019). Plant-parasitic nematodes, however, are rarely indicated as a threat, or as a cause for concern even. There is in general, an almost complete lack of knowledge of nematodes as pests of coffee within the coffee sector in East Africa (Souza, 2008; Coyne et al., 2018; Villain et al., 2018). This is despite being considered amongst the most serious production constraints of coffee in other parts of the world, such as in Latin America and Asia (Souza, 2008; Villain et al., 2018).

In Africa, this declining trend in coffee production is particularly evident in Kenya, where coffee has traditionally been one of the country’s most important export crops (Coffee Research Institute - Kenya Agricultural and Livestock Research Organization, 2022). Although demand for Kenyan coffee remains high on the global market, due to the high quality of the Arabica type primarily grown, investments in new plantations are limited, with yields sliding in the ageing plantations. With perennial crops, pest and disease issues often build up gradually over time, especially nematode pests (Campos and Silva, 2008). The hallmark of nematode infection in mature plantations is the gradual suppression of growth and productivity over time, without necessarily causing plant death. Nematodes are especially aggressive on transplanted young seedlings, while on mature plants they can induce nutrient deficiencies, defoliation, stunting and ultimately impact yield (Villain et al., 2018). Above-ground symptoms of nematode damage, however, are notoriously difficult to diagnose, as they are otherwise indistinguishable from general plant malaise and stunting due to low soil fertility, nutrient deficiencies or low water availability and drought susceptibility (Coyne et al., 2018). Considering the damage that nematodes are known to cause on coffee in Latin America and Asia, the likelihood that they are also imposing a sizable damage on Kenyan coffee production is high.

Globally, root-knot nematodes (RKN; *Meloidogyne* spp.) are viewed as the number one nematode pest across crops (Jones et al., 2013) and considered as the greatest biotic threat to agriculture in sub-Saharan Africa (Coyne et al., 2018). On coffee, they are also the most commonly occurring and are generally regarded as the most important nematode pest (Villain et al., 2018).

There are several RKN species that infect coffee, as well as other genera of plant-parasitic nematodes (Humphreys-Pereira et al., 2014; Nzesya et al., 2014; Janssen et al., 2016; Gitonga, 2020). Among the coffee-parasitic RKN species, 17 have been recognized worldwide: *M. exigua, M. Africana, M. arabicida, M. arenaria, M. coffeicola, M. decalineata, M. hapla, M. incognita, M. inornate, M. izalcoensis, M. javanica, M. kikuyensis, M. konaensis, M. mayaguensis, M. megadora, M. oteigae* and *M. paranaensis* (Carneiro and Cofcewicz, 2008). Nematologists and extensionists must identify the species of parasitic nematodes infesting a coffee field at the species level in order to determine the appropriate management strategies, especially with regards to which resistance sources should be employed (Campos and Silva, 2008).

In East Africa, the information on species distribution and incidence in the region is scant and erratic, with most records documenting their detection only. Data on the levels of damage nematodes cause to coffee in the region, or their economic importance, is effectively non-existent and yet to be determined. Coffee yield losses due to nematode infection is based mostly on data from Latin America and documented as between 10 to 35%, depending on species (Barriga, 1976; Bertrand et al., 1997; López-Lima et al., 2015), but with some indications of wholescale plantation destruction in Brazil (Lordello, 1984). Considering the perennial nature of coffee, however, accurately estimating the economic losses due to nematodes is difficult. In addition, loss estimates should also consider the impact of infection on plantation longevity and the replant cost of non-performing trees.

The management of soil-borne pests and diseases on perennial crops presents numerous challenges. Once established, they become extremely difficult to eliminate, including nematodes. Reducing infection through the use of clean, healthy, resistant and protected seedlings is therefore an advised nematode management strategy, and a policy which is being actively promoted in Latin America and Asia (Villain et al., 2018). In Africa, with an almost total ignorance of coffee nematode pests, few if any management practices are observed or followed. With limited recognition and costly or unsuitable management options available, such as total renovation of aged plantations, the outlook does not look positive. Smallholder farmers also represent a high proportion of coffee farmers in the region, who are less able or flexible to incorporate nematode management practices.

In addition to the practical difficulties of nematode management in coffee fields, sustainability aspects also need to be taken into consideration. Nematicides are often a first line of defense, especially in more commercial farming systems. Biological control options, however, offer environmentally sensitive alternatives, especially under organically oriented systems. And in regard to this, there is increasing interest in the use of biologically based products for pest and disease management and improving coffee production (Alwora and Gichuru, 2014). Various microbial antagonists have been identified for use on coffee, such as the bacterium *Pasteuria penetrans* (Sharma and Lordello, 1992; Maximiniano et al., 2001) and egg parasitic fungi (Naves and Campos, 1991; Ribeiro and Campos, 1993). *Trichoderma* spp. is the most studied biocontrol fungi and has been extensively investigated and utilized for their capacity to compete and parasitize phytopathogens as well as to mitigate unfavorable growth conditions (Poveda, 2021). *Purpureocillium lilacinum* (=*Paecillomyces lilacinus*) is also a well-recognized fungal biocontrol agent for use against root-knot nematodes (Holland et al., 1999; Kiewnick and Sikora, 2006; Heidari and Olia, 2016). On coffee, Campos and Campos (1997) demonstrated the potential of *P. lilacinum* against *Meloidogyne exigua* on seedlings in Brazil. Additionally, *Trichoderma harzianum* (Arita et al., 2020; Zinger et al., 2021) and *P. lilacinum* (Arita et al., 2020) have been evaluated for efficacy against RKN on coffee in Brazil. Although they were ineffective against the nematodes, *P. lilacinum* promoted coffee plant growth (Arita et al., 2020). In Kenya, both *P. lilacinum* and *Trichoderma asperellum* are registered for use against nematodes and are commercially available, where they have been proven effective against RKN on pineapple (Kiriga et al., 2018) and *T. asperellum* against *Radopholus similis* on banana (Kisaakye et al., 2023).

Given the current scarcity of suitable or sustainable nematode management options amongst a rising global demand for nematode control, there is much current interest to identify options including biologically based alternatives to synthetic chemicals (Campos and Silva, 2008; Coyne et al., 2018). However, how the long-term and intensive application of biological products to perennial crops impacts on the soil food web and soil ecosystem functions has been little studied. Some studies have demonstrated the positive effect of *Trichoderma* spp. on enhancing the species richness and evenness of microbial distribution (Alexander and Phin, 2014) as well as the improved availability of nutrients for microbial consumption and ultimately promotion of plant health (Cai et al., 2015; Carvalhais et al., 2015). Conversely, some studies have indicated that application of microbial products disrupts the microbial community structure in the rhizosphere leading to impaired nutrient absorption (Ros et al., 2017; Halifu et al., 2019). Free-living nematodes have long been recognized as useful bioindicators of soil health due to their high abundance, rapid response to new resources and food specificity (Yeates et al., 1993). Since the development of nematode community indices (Bongers, 1990; Ferris et al., 2001; Ferris and Bongers, 2009), attention towards the use of nematodes as a quantitative measure to evaluate the impact of management practices on soil health (Okada and Harada, 2007) and measure the ecological impact (Krashevska et al., 2019) has increased.

This current study evaluated the application of two commercial products based on *P. lilacinum* (IMPEDE^®^, RealIPM, Kenya) and *T. asperellum* (SUSTAIN^®^, RealIPM, Kenya) over an 18-month period on Kenyan Arabica coffee suffering from *Meloidogyne* infection. The study focused on in-field application to established coffee fields of various ages. The objectives were to evaluate the efficacy of *T. asperellum* and *P. lilacinum* to suppress field densities of plant-parasitic nematodes, and to evaluate these products for their impact on the soil food web and soil health using nematode community assemblages as a basis.

## 2 Materials and methods

### 2.1 Study sites and experimental design

The field trials were conducted on Chania Estate, Kiambu County, Kenya (1°01’36”S, 37°01’20”E). The study comprised trials in seven Arabica coffee fields, three with cv. Ruiru 11 and four with cv. French Mission. Fields were planted between 1926 to 2012 and the age varied by field. Two fields were planted in 1926, three were planted in 2012, and the rest were planted in 1960 and 1992. The coffee plants are pruned after every harvest. The average temperature and rainfall ranged from 20 °C (May in 2021) to 24 °C (October in 2020) and from 0 mm (June, September in 2021, January in 2022) to 230.8 mm (April in 2021). The fields received no irrigation. In each field, three treatment plots were demarcated, each consisting of four replications of five trees each, arranged in a line, giving 12 experimental units per field and 84 experimental units in total. The three treatments included *T. asperellum, P. lilacinum* and untreated control. Treatments were applied around the base of each tree after creating a ~1 m diam. basin with a hand hoe, which helped contain the applied microbial suspension to the tree base. Treatments were consistently applied using the same volumes on each occasion: 20 L of *T. asperellum* suspension (2x10^7^ cfu/L), 20 L of *P. lilacinum* suspension (2x10^7^ cfu/L) according to the manufacturer’s recommendation and 20 L water (control). The regularity of these applications reduced over time. For the first month, treatments were applied weekly to initially immerse the root system with the products and to encourage the fungal colonization in soil and roots. From the second month, treatments were applied bi-weekly for 11 months until fungal colonization was confirmed in roots. From 12 months onwards, treatments were applied monthly for six months. The field trial was conducted over a total of 25 months from February 2020 to March 2022. For the latter seven months of the study, half of each plot in each field received no treatment application to evaluate the treatment effect persistence in soil and roots.

### 2.2 Data collection

Soil and root samples were collected from each trial site prior to the beginning of the study. Data were not collected for the following six months due to Covid-19 restrictions. Thereafter, soil samples were collected from each experimental unit monthly and root samples every six months, except for the final root sampling, which was collected eight months after the previous sampling. Samples were collected from the root zone of each of the five trees per replication for soil and three trees per replication for the newly developed lateral roots from 5 to 20 cm depth and combined for each replication. Soil and root samples were processed separately using the modified Baermann plate method for nematode extraction from 100 cm^3^ soil and 5 g fresh root sub-samples (Coyne et al., 2014; Saikai et al., 2021). All plant-parasitic nematodes were identified to genus level and counted at 40X magnification using a ZEISS Primo Star binocular microscope (Carl Zeiss AG, Oberkochen, Germany) for all monthly samples. *Meloidogyne* spp. infecting roots were further identified to species level morphologically, based on the perineal patterns of mature females, and molecularly, based on COI mtDNA (Janssen et al., 2016). Every second month, free-living nematodes in the samples were identified to genus level and counted at 40X or 100X magnifications from the first 100 encountered nematode individuals in each sample. When nematodes were unable to be assigned to genus level morphologically, they were assigned to family level (i.e. Cephalobidae, Rhabditidae and Tylenchidae). The nematodes were assigned to their trophic groups according to Yeates et al. (1993). The total abundance of each genus and each trophic group were calculated based on the total abundance of all the nematodes and the abundance of each genera/trophic group in 100 identified nematodes. These data were uploaded to NINJA (http://spark.rstudio.com/bsierieb/ninja/; Sieriebriennikov et al., 2014) to calculate the footprints, the relative abundance of total or free-living nematodes for each trophic group and each colonizer-persister group (c-p group), as well as nematode indices (i.e. Basal Index, Channel Index, Enrichment Index, Maturity Index, Maturity Index 2-5, Sigma Maturity Index and Structure Index) for each of the data entries. On a six-monthly basis, plant-parasitic nematodes were assessed from the roots and fungal colonization assessed from soil and root samples. Colony forming units of *T. asperellum* and *P. lilacinum* were quantified from ~5 g fresh root and 1 g soil sub-samples using the spread plate culture technique (Hohmann et al., 2011) on Potato Dextrose Agar with Acid.

### 2.3 Data analysis

Nematode data from root samples were analyzed using the generalized linear mixed model (PROC GLIMMIX, SAS Institute, Cary, NC) to evaluate the effect of treatments on nematodes infecting roots at each of the five different time points. Studentized residual plots were generated by PLOTS = STUDENTPANEL. The Satterthwaite approximation was used to adjust the degrees of freedom. Appropriate distributions were selected for each of the response variables after comparing the diagnostic plots and model fitness for preliminary model runs using multiple distributions. Effects of cultivar, field nested within cultivar, and replication nested within cultivar and field were included as random (G-side) effects. Tests of covariance parameters using the Restricted Likelihood were conducted for each combination of the random effects using COVTEST option. The effect of treatment was compared by performing the mean separation test using the least square means. For the root data for March 2022, in addition to the model to assess differences between treatments in the fixed (R-side) effect, an additional generalized linear mixed model was performed, which included the binary variable for trees that were treated or not treated, in addition to the treatment effect, and the interaction of the two. The remainder of the model options remained the same.

The soil samples were analyzed separately for samples before the fungal isolation from roots (Oct. 2020~Feb. 2021: 4 time points) and after isolation (Mar. 2021~Apr. 2022: 11 time points). Each time set of the data points was analyzed using the generalized linear mixed model (PROC GLIMMIX, SAS Institute, Cary, NC). The same options as the models for the root samples were applied for the residual plots and the degrees of freedom adjustment. The effect of treatment, time and the interaction of treatment and time on the parasitic nematode density in soil were included as fixed effects. The effects of cultivar, field nested within cultivar, were included as random and the interaction of replication, cultivar, field and treatment were added in the random side of the model as a whole plot error. Time was treated as a repeated measure. The first order autoregressive covariance structure was selected after evaluating the model fitness using other covariance structures in the preliminary model runs and based on the fact that these different time point data were collected at equally spaced time points. The effect of treatment was compared at each time point by performing a partitioned analysis of the least square means with *p-*value adjusted by the Tukey procedure.

Pearson correlation coefficient was also computed between the nematode density in soil and in roots using cor() function of the basic package in R (R Core Team, 2020). The regression analysis was performed between the nematode density in soil and roots after transforming by log10(N+1) for both variables to satisfy the model assumptions using lm() function of the basic R package.

Similar to the assessment of soil samples for parasitic nematodes, the nematode community data were analyzed separately for samples before fungal isolation from roots (Oct. 2020~Feb. 2021: 3 time points) and after isolation (Apr. 2021~Apr. 2022: 7 time points) to evaluate the effects of the treatments on the abundance parameters and nematode indices that were computed by NINJA using the same model and model options used to analyze the soil samples.

Principle component analysis (PCA) was performed separately on the early (Oct. 2020) and on the final nematode community data (Apr. 2022) using *facto extra* package. The 10 most common genera detected were included in the analysis, with the less common genera omitted from analysis. Before the analysis, the nematode counts were standardized to have mean=0 and standard deviation=1 for each genus at the individual time points. PCA values and associated Eigenvalues were computed by prcomp() and get_eigenvalue() functions in the package. The bi-graph was generated for the individuals (i.e. the nematode genera) and the vectors (i.e. the treatments) by fviz_pca_biplot() function.

Functional metabolic footprints of nematodes in the soil food webs were visualized by plotting Structure index on X-axis against Enrichment index on Y-axis. The visualized plots were interpreted according to Ferris et al. (2001) and Ferris (2010).

## 3 Results

### 3.1 Effects of biocontrol agents on *Meloidogyne* spp. densities

All seven fields used in the study were infested with RKN, which was identified as *Meloidogyne hapla.* This was the most dominant nematode genus throughout the study period, especially from roots with densities of second stage infective juveniles (J2) ranging between 0-1,828 per 100 cm^3^ soil and 0-3,492 per 5 g roots. Other parasitic nematodes (*Helicotylenchus* spp. (0-20 per 100 cm^3^ soil), *Hemicycliophora* spp. (0-5 per 100 cm^3^ soil), *Trichodorus* spp. (0-213 per 100 cm^3^ soil), *Tylenchorhynchus* spp. (0-4 per 100 cm^3^ soil), *Pratylenchus* spp. (0-19 per 100 cm^3^ soil), and *Rotylenchulus* spp. (0-766 per 100 cm^3^ soil)) were also recovered but their distribution was sporadic and in general were not prevalent.

**Figure 1:**
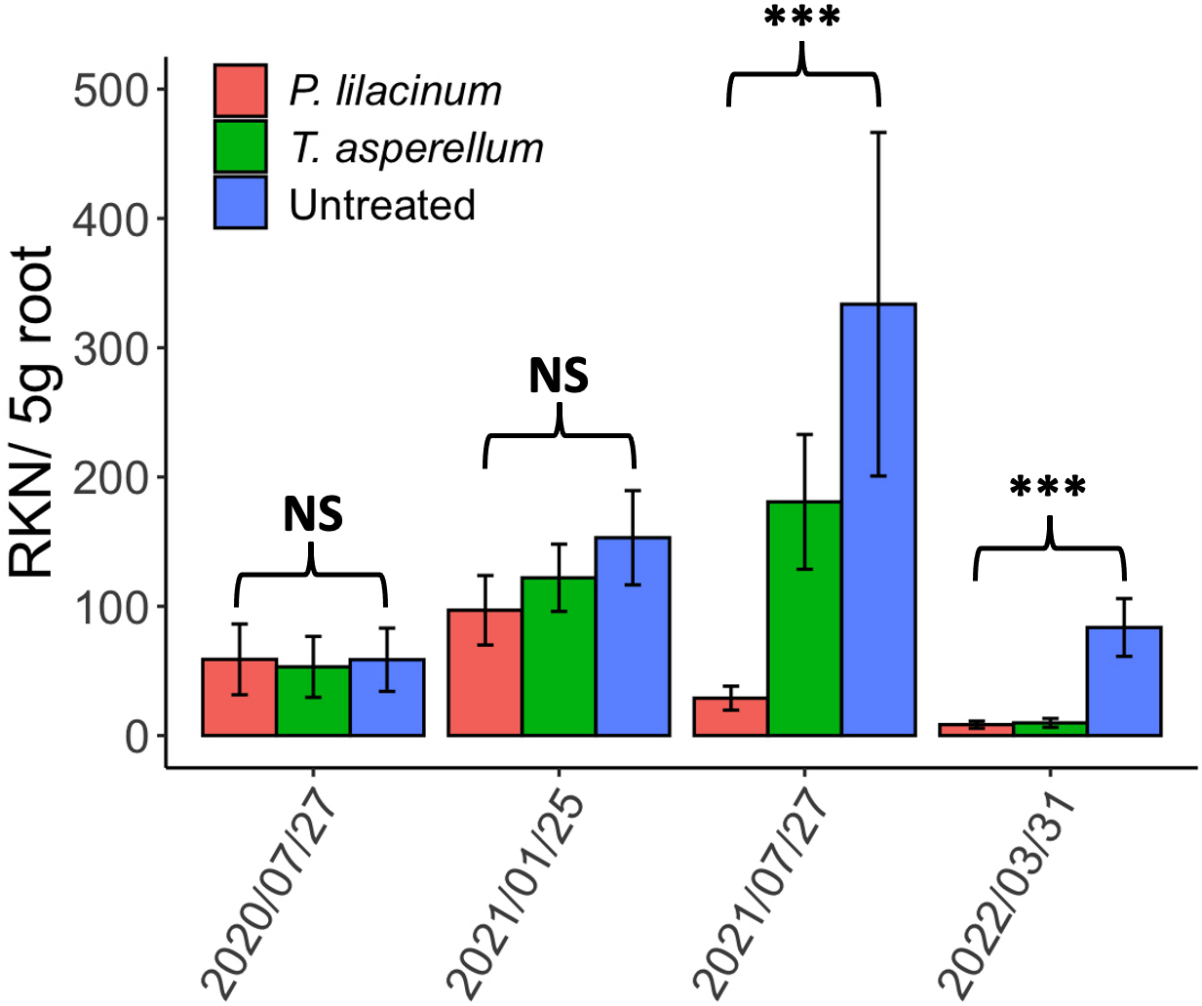
The bar plot of *Meloidogyne hapla* in 5g roots from August 2020 to April 2022 for *T. asperellum, P. lilacinum* and the untreated. Asterisks represent the statistically significant differences among the treatments. NS, not significant.

No effect of either biocontrol agent was observed on the RKN root density until 2021 August, six months after first detecting fungal colonization in roots (**Figure 1**). RKN root densities were lower in plots treated with either biocontrol agent on 2021 August and on 2022 April sampling (*P* ≤ 0.05) than in the control plots. Densities were lower in *P. lilacinum* treated plots than *T. asperellum* plots on 2021 August (*P* ≤ 0.05) but RKN root densities between the two biocontrol agents were similar for the 2022 April samples (**Figure 1**).

The tests of covariance parameters based on the restricted likelihood demonstrated the significant contribution of the cultivar effect in G-side effects (*P* ≤ 0.05) for the 2021 August sampling but not for the 2022 April sampling. Other random effects, such as tree age, field, and replication, were not significant. When the cultivar effect and the interaction between the effects of cultivar and treatment were included in the R-side of the generalized linear mixed model, both the cultivar effect and its interaction with the treatment effect were insignificant without alternating the data interpretation of the treatment effect above. This confirms that both ‘French mission’ and ‘Ruiru 11’ are equally susceptible to *M. hapla* and react similarly to the biocontrol agents. *Rotylenchulus* spp. was sporadically found cohabiting with RKN in roots in low to medium relative densities (mean=6.8, range 0 to 256.9/5 g root) but no significant effects of the biocontrol agents were observed on these nematodes.

The RKN soil density was not affected by treatment with either biocontrol agent, except for the 2021 March samples, when densities were lower in *P. lilacinum* treated plot soil than in the untreated and *T. asperellum* plots (*P* ≤ 0.05). The correlation coefficient between the RKN soil density and the root density was 0.30 and the relationship was positive between the log (base 10)-transformed densities in soil and roots (*P* ≤ 0.01). Based on the regression model, a unit increase in RKN soil density will result in an increase in RKN root density by 0.13, although just 8% of the variation in RKN root density can be explained by the soil densities based on adjusted R^2^.

### 3.2 Fungal colonization of soil and roots

The fungal colonization in soil and roots were confirmed 6 months and 12 months after the initial treatments, respectively. The fungal soil colonization for both treatments fluctuated by season and ranged from 1.26 x 10^2^ (Aug. 2021) to 3.22 x 10^3^ cfu/g soil (Feb. 2022) for *P. lilacinum* and 1.38 x 10^2^ (Aug. 2021) to 2.45 x 10^6^ cfu/g soil (Feb. 2022) for *T. asperellum*, which were unaffected by the tree age (*P* > 0.05). There was no difference observed in fungal colonization of *T. asperellum* treated roots and the soil during the final 8 months between the plots that received the application and the plots that did not (*P* > 0.05). Conversely, the *P. lilacinum* colonization in soil became undetectable (0 cfu/g soil) in five of the seven fields. The other two fields remained colonized at the same level between plots with and without the application, although this did not seem to correlate to age or cultivar. For both biocontrol agents, the fungal colonization in roots at the final sampling (Feb. 2022) was significantly lower (*P* ≤ 0.05) than the previous sampling (Aug. 2021), where *P. lilacinum* had 2.23 x 10^4^ cfu/g root and *T. asperellum* had 1.98 x 10^4^ cfu/g root. In the final sampling, the fungal colonization in roots decreased to 7.0 x 10^-1^ cfu/g root for *P. lilacinum* on plots received no application and 5.29 x 10^2^ cfu/g root for *T. asperellum* without no significant difference between plots with and without applications. The effect of *T. asperellum* persisted in coffee roots for at least eight months after the final application given that there was no significant difference in the RKN root density between with and without applications.

### 3.3 Effect of biocontrol agents on the soil nematode community

In total, 59 genera and family of free-living nematodes were recovered from soil samples over the two years of study (**Table 1**). The ten most abundant nematode genera, in descending order, were: *Cephalobus*, *Pseudacrobeles, Aphelenchus, Aphelenchoides, Meloidogyne, Prismatolaimus, Rotylenchulus, Mesorhabditis, Tylenchidae, Panagrellus.* Total mean abundance was 51.2% (38.3 - 63.9%) for bacterivores, 30.9% (16.7 - 49.3%) for fungivores, 16.0% (8.9 - 27.0%) for herbivores and 6.9% (2.5 - 12.0%) for omnivores and predators.

**Table 1:** The mean and range of the total abundance of nematodes in soil for each genus.

Nematode genus/family	*P. lilacinum*	*T. asperellum*	Untreated
Bacterivores:			
*Acrobeles*	1.91 (0-67.4)	1.60 (0-28.2)	1.40 (0-27.7)
*Acrobeloides*	0.30 (0-19.3)	0.53 (0-20.7)	0.41 (0-16.5)
*Alaimus*	4.76 (0-176.5)	2.31 (0-76.0)	2.65 (0-76.9)
*Alloionematidae*	0.03 (0-4.9)	0.05 (0-13.1)	0.03 (0-6.7)
*Cervidellus*	1.99 (0-26.7)	1.93 (0-51.6)	2.16 (0-34.0)
*Chronogaster*	1.92 (0-37.1)	3.05 (0-93.7)	3.41 (0-59.0)
*Cryptonchus*	0.29 (0-17.7)	0.75 (0-116.0)	0.55 (0-106.5)
Cephalobidae	60.60 (0-459.9)	67.52 (3.0-450.9)	78.74 (3.5-406.0)
*Diplogaster*	0.22 (0-9.8)	0.59 (0-64.6)	0.32 (0-28.3)
*Eumonhystera*	0.27 (0-18.2)	0.72 (0-105.8)	0.69 (0-45.0)
*Geomonhystera*	2.02 (0-88.3)	2.38 (0-59.7)	2.45 (0-70.1)
*Panagrellus*	15.13 (0-3220.6)	3.35 (0-141.5)	2.98 (0-77.3)
*Panagrolaimus*	5.59 (0-66.9)	4.82 (0-69.7)	5.58 (0-172.1)
*Plectus*	1.78 (0-26.7)	2.06 (0-32.8)	2.37 (0-172.1)
*Pseudacrobeles*	48.36 (0-334.5)	49.20 (0-269.2)	63.48 (0-1228.8)
*Prismatolaimus*	10.29 (0-133.5)	10.47 (0-78.3)	11.06 (0-152.1)
*Protorhabditis*	0.99 (0-105.6)	0.99 (0-67.9)	1.25 (0-103.1)
*Rhabditis*	0.75 (0-19.6)	1.38 (0-63.1)	1.68 (0-41.5)
Rhabditidae	0.08 (0-10.3)	0.03 (0-5.6)	0.05 (0-9.2)
*Tobrilus*	2.42 (0-54.0)	2.08 (0-48.0)	2.21 (0-55.3)
*Metacrobeles*	0.05 (0-6.2)	0.11 (0-7.8)	0.02 (0-3.4)
*Mesorhabditis*	8.73 (0-87.6)	7.60 (0-103.7)	12.33 (0-172.6)
*Wilsonema*	0.95 (0-24.4)	1.16 (0-36.8)	0.96 (0-28.2)
Fungivores:			
*Aphelenchus*	58.51 (0-480.9)	45.10 (0-284.5)	44.92 (0-249.7)
*Aphelenchoides*	48.45 (0-627.2)	42.93 (0-464.5)	38.46 (0-368.7)
*Diphtherophora*	0.59 (0-20.7)	0.56 (0-13.3)	0.52 (0-9.1)
*Filenchus*	3.43 (0-45.9)	4.63 (0-54.7)	4.97 (0-92.9)
Herbivores:			
*Ecphyadophora*	0.12 (0-7.9)	0.10 (0-6.6)	0.02 (0-8.0)
*Helicotylenchus*	0.05 (0-3.1)	0.31 (0-19.6)	0.34 (0-35.9)
*Hemicycliophora*	0.06 (0-4.2)	0.03 (0-2.8)	0.08 (0-11.7)
*Pratylenchus*	0.06 (0-8.0)	0.02 (0-2.9)	0.09 (0-6.9)
*Paratrichodorus*	0.17 (0-18.2)	0.09 (0-10.6)	0.23 (0-17.8)
*Psilenchus*	0.05 (0-5.1)	0.04 (0-3.7)	0.05 (0-8.6)
*Rotylenchulus*	11.47 (0-265.9)	12.09 (0-444.2)	6.35 (0-323.3)
*Scutellonema*	0.02 (0-1.9)	0.02 (0-4.8)	0.00
*Tylenchorhynchus*	0.01 (0-2.1)	0.03 (0-3.9)	0.003 (0-1.0)
*Trichodorus*	3.34 (0-45.2)	4.62 (0-106.5)	4.49 (0-147.0)
*Tylenchus*	4.50 (0-58.9)	6.43 (0-132.0)	9.35 (0-504.0)
Tylenchidae	8.06 (0-206.8)	6.88 (0-153.2)	8.24 (0-225.7)
*Meloidogyne*	50.28 (0-1639.6)	39.79 (0-1816.2)	33.77 (0-1159.4)
*Paratylenchus*	0.004 (0-1.3)	0.06 (0-9.2)	0.06 (0-16.9)
Predators & Omnivores			
*Anatonchus*	0.02 (0-4.6)	0.04 (0-5.7)	0.04 (0-7.7)
*Axonchium*	0.01 (0-2.1)	0.03 (0-5.6)	0.01 (0-3.5)
*Aporcelaimellus*	0.09 (0-10.4)	0.04 (0-4.9)	0.08 (0-7.5)
*Clarkus*	0.50 (0-16.1)	0.62 (0-27.5)	0.72 (0-17.6)
*Dorylaimus*	0.21 (0-11.2)	0.45 (0-13.4)	0.37 (0-14.5)
*Discolaimus*	0.58 (0-24.7)	0.47 (0-12.0)	0.71 (0-17.8)
*Eudorylaimus*	2.36 (0-45.3)	2.95 (0-54.9)	2.01 (0-52.6)
*Epidorylaimus*	0.08 (0-10.8)	0.11 (0-12.4)	0.08 (0-8.0)
*Labronema*	0.01 (0-2.3)	0.008 (0-2.2)	0.09 (0-22.6)
*Pristionchus*	0.17 (0-37.1)	0.07 (0-9.3)	0.16 (0-18.0)
*Prodorylaimus*	4.91 (0-85.8)	6.57 (0-105.2)	4.07 (0-171.8)
*Thonus*	3.68 (0-39.8)	4.72 (0-42.7)	3.02 (0-32.5)
*Tripyla*	1.60 (0-56.1)	2.23 (0-83.7)	3.11 (0-94.6)
*Mesodorylaimus*	2.53 (0-39.3)	3.55 (0-77.4)	1.88 (0-46.3)
*Miconchus*	0.07 (0-10.3)	0.06 (0-11.9)	0.01 (0-3.6)
*Microdorylaimus*	0.35 (0-28.9)	0.23 (0-26.9)	0.33 (0-15.2)
*Mylonchulus*	0.43 (0-25.0)	0.88 (0-14.8)	0.69 (0-10.5)

The generalized linear mixed model with time as a repeated measure revealed that application of the biocontrol agents before the fungal isolation from roots had no significant effect on the nematode indices (**Table 2**).

**Table 2:** The *p-*values of the effects of treatment, time and their interaction from models based on the data points before fungal isolation from roots and after fungal isolation from roots for nematode community parameters.

		Before fungal isolation from roots				After fungal isolation from roots			
Parameters	Distribution	n	Treatment	Time	Treatment x Time	n	Treatment	Time	Treatment x Time
**Basal index**	gaussian	260	NS	0.05	NS	596	0.0008	<0.0001	0.025
**Channel index**	gaussian	260	NS	0.0003	NS	596	0.017	<0.0001	0.003
**Enrichment index**	gaussian	260	NS	0.03	NS	596	0.03	<0.0001	0.007
**Maturity index**	gaussian	260	NS	<0.0001	NS	596	0.002	<0.0001	NS
**Maturity index 2-5**	gaussian	260	NS	<0.0001	NS	596	0.002	<0.0001	NS
**Plant-parasitic index**	gaussian	257	NS	NS	0.005	583	NS	<0.0001	NS
**Sigma maturity index**	gaussian	260	NS	0.07	NS	596	0.003	<0.0001	NS
**Structure index**	gaussian	260	NS	<0.0001	NS	596	0.003	<0.0001	NS
**Shannon index**	gaussian	260	NS	<0.0001	NS	596	0.02	<0.0001	NS
**Bacterivore footprint**	log-normal	260	0.0001	<0.0001	NS	596	NS	<0.0001	NS
**Fungivore footprint**	log-normal	260	NS	<0.0001	NS	596	<0.0001	<0.0001	NS
**Herbivore footprint**	log-normal	257	NS	<0.0001	NS	583	NS	<0.0001	NS
**Omnivore footprint**	log-normal	181	0.08	0.03	NS	472	0.04	<0.0001	NS
**Predator footprint**	log-normal	110	0.03	0.0008	NS	317	NS	<0.0001	NS
**% Bacterivores in free-living nematodes**	gaussian	260	0.001	<0.0001	NS	596	<0.0001	<0.0001	0.05
**% Bacterivores in total nematodes**	gaussian	260	0.0003	0.003	NS	596	<0.0001	<0.0001	NS
**% Fungivores in free-living nematodes**	gaussian	260	0.003	<0.0001	NS	596	<0.0001	<0.0001	0.08
**% Fungivores in total nematodes**	gaussian	260	NS	<0.0001	NS	596	<0.0001	<0.0001	0.05
**% Herbivores in total nematodes**	log-normal	257	NS	0.0003	NS	583	NS	<0.0001	NS
**% Omnivores in free-living nematodes**	log-normal	181	NS	<0.0001	NS	472	0.001	<0.0001	NS
**% Omnivores in total nematodes**	log-normal	181	NS	<0.0001	NS	472	0.001	<0.0001	NS
**% Predators in free-living nematodes**	log-normal	110	NS	NS	0.08	317	NS	<0.0001	NS
**% Predators in total nematodes**	log-normal	110	NS	0.06	NS	317	NS	<0.0001	NS
**Fungivores: Bacterivores**	log-normal	260	NS	0.002	NS	596	<0.0001	<0.0001	NS
**% c-p 1 in free-living nematodes**	log-normal	247	NS	<0.0001	NS	554	NS	0.0002	0.06
**% c-p 2 in free-living nematodes**	log-normal	260	NS	0.0003	NS	596	0.07	<0.0001	0.02
**% c-p 3 in free-living nematodes**	log-normal	222	NS	<0.0001	0.02	496	0.07	<0.0001	NS
**% c-p 4 in free-living nematodes**	log-normal	205	NS	<0.0001	NS	488	0.003	<0.0001	NS

NS, not significant.

The bacterivore footprint was larger for the control treatment, before fungal isolation from roots (*P* = 0.0001), whereas the difference in the footprint became insignificant following fungal isolation. However, the relative abundance of bacterivores in the nematode community (% bacterivores of total nematodes) and of free-living nematodes (% bacterivores of free-living nematodes) remained significant after the fungal isolation (**Table 2**). The relative abundance of bacterivores in the free-living nematode community had a significant interaction between the treatment and time, in which the two biocontrol agents occasionally had similar relative abundance of bacterivores. The control treatment had the highest and *P. lilacinum* had the lowest values for the relative abundance of bacterivores in free-living nematode community. Conversely, the relative abundance of fungivores in the free-living nematode community (% fungivores of free-living nematodes) was greater for *P. lilacinum* than the other treatments (*P* = 0.003), which was not affected by the sampling time points.

The fungivore footprint was not affected by treatment before fungal isolation from roots but it significantly increased for *P. lilacinum* after (*P* < 0.0001) (**Table 2**). The relative abundance of fungivores in both free-living and total nematode communities was consistently high for *P. lilacinum* among the three treatments, although there were occasionally significant differences for the other two treatments with the untreated generally having the lowest values. *Purpureocillium lilacinum* had a lower omnivore (*P* = 0.08) and predator footprint (*P* = 0.03), relative to the other two treatments, before the fungal isolation (**Table 2**). The omnivore and predator footprints were lower for *P. lilacinum* before fungal isolation (*P <*0.1). However, a higher omnivore footprint was associated with both biocontrol agents after fungal isolation (*P* = 0.04) but not for the predator footprint.

The models based on the soil samples after the fungal isolation from roots demonstrated that the Maturity index, Maturity index 2-5 and Sigma maturity index, structure index, and Shannon index all significantly increased for *T. asperellum* compared to the control, while for *P. lilacinum* they did not differ from *T. asperellum* or the control. Before fungal isolation, none of the treatments had any effect on these nematode based indices (**Table 2**). Although the treatment effect was significant for basal, channel and enrichment indices after the fungal isolation, there was no consistency in how treatments influenced the indices (**Table 2**). The ratio of fungivores to bacterivores significantly increased for *P. lilacinum* after the fungal isolation from roots. There was no significant effect of either biocontrol agent on any of the c-p groups before the fungal isolation in roots, however, *T. asperellum* increased the number of nematodes belonging to c-p 3 and the c-p 4 groups after the fungal isolation (**Table 2**). The number of nematodes in c-p 2 was also significantly affected by the treatment but inconsistently after the fungal isolation, indicated by the significant interaction between the treatment and the time effects. Both the abundance of *Cephalobus* and *Pseudocrobeles* were greater for the control than for *P. lilacinum* after the fungal isolation in roots (*P* ≤ 0.05) but not affected before (*P* > 0.1). Interestingly, *Aphelenchus* density was significantly higher for *P. lilacinum* after the fungal isolation (*P* ≤ 0.0001), whereas *Aphelenchoides*, another dominant fungivore in samples, was unaffected. Other abundant genera were not affected by the treatment before nor after the fungal isolation. The time effect was significant for all the parameters except for the plant-parasitic index and the abundance of predators in the free-living nematode community before fungal isolation from roots, indicating that these values fluctuate significantly with time.

The footprint of all the trophic groups, the relative abundances in total and in free-living nematodes for herbivore, omnivore and predator, and the relative abundances in free-living nematodes of all the c-p group fitted lognormal distributions to satisfy the model assumptions, especially homoscedasticity. Other parameters were described by Gaussian distribution. The covariance test on the random effects revealed that the cultivar effect was significantly contributing in the G-side. The exceptions were the channel index, the enrichment index, the relative abundances of fungivores and predators in total nematodes and free-living nematodes before fungal isolation from roots, and the abundance of c-p 1 group in free-living nematodes before and after fungal isolation, which had no significant effect in G-side. The predator footprint and the ratio of fungivores to bacterivores before fungal isolation and the channel index after the fungal isolation had a significant contribution of the replication but not for the field or variety in G-side. The relative abundances of fungivores and herbivores in the total nematode community after the fungal isolation from roots demonstrated that all the random effects in G-side were significant.

**Figure 2:**
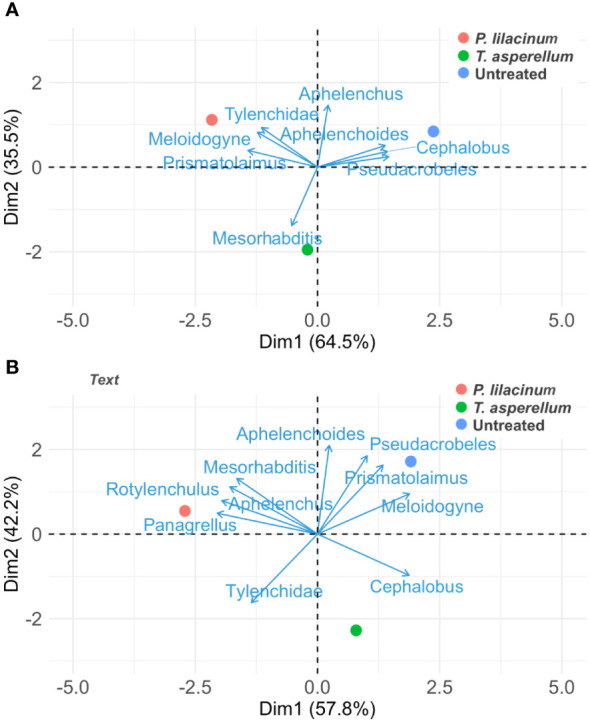
Principal component analysis of the treatments as related to nematode genera in the early nematode community data sampled on Oct. 2020 **(A)** and the final nematode community data sampled on Apr. 2022 **(B)**.

PCA on the early (Oct. 2020) and final nematode community data (Apr. 2022) revealed distinguished nematode community profiles of the biocontrol agents, with significant changes in nematode genera associated with each of the treatments between the time points (**Figures 2A, B**). The Oct. 2020 nematode community data showed strong associations with *Cephalobus*, *Aphelenchoides*, *Pseudacrobeles* for the untreated, Tylenchidae, *Meloidogyne* and *Prismatolaimus* for *P. lilacinum*, and *Mesorhabditis* for *T. asperellum* (**Figure 2A**). The Apr. 2022 nematode community data, however, revealed a strong association of *Pseudacrobeles, Prismatolaimus* and *Meloidogyne* with the control and *Mesorhabditis, Aphelenchus, Rotylenchulus* and *Panagrellus* with *P. lilacinum* (**Figure 2B**). The first and second principal components explained 64.5% (Eigenvalue = 5.18) and 35.5% (Eigenvalue = 2.84) of the total variance of the Oct. 2020 nematode community data, respectively. For the Apr. 2022 nematode community data, the first principal component explained 57.8% (Eigenvalue = 4.22) and the second principal component explained 42.2% (Eigenvalue = 4.22) of the total variance.

**Figure 3:**
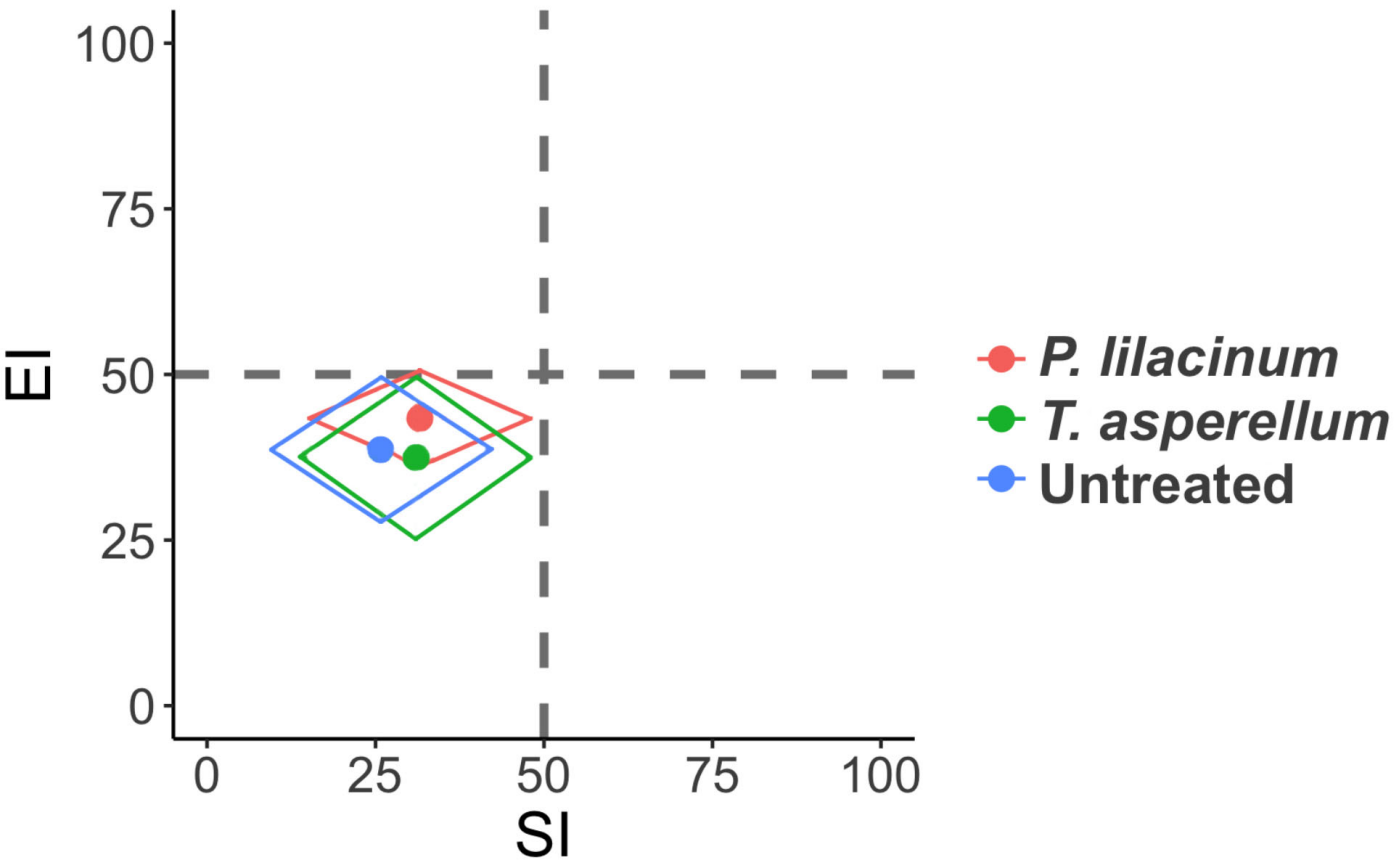
Functional metabolic footprints of nematodes with different treatments. Vertical axis represents the enrichment footprint and horizontal axis the structure footprint.

Functional metabolic footprints demonstrated no separation among the treatments during the two years of the field trial (**Figure 3**). The three treatments all clustered to the left bottom quadrant, inferring a degraded food web condition and soils that are stressed, depleted, fungal decomposition channels, and a high C:N ratio.

## 4 Discussion

Over a two-year period, the application of *T. asperellum* and *P. lilacinum* products demonstrated that even over this relatively short period, RKN densities, and consequently damage, could be suppressed on established Arabica coffee plantations. Some positive changes in the soil food web structure were also observed following the application of these biocontrol agents. How sustainable this is, in terms of reversing such damage, yet needs to be determined, as does the economic viability of such a practice. But this study demonstrates the potential for the use of biocontrol agents on perennial crops. It took up to one year before we detected endophytic colonization of the fungi in coffee roots, which was longer than we anticipated. However, this could have been affected by tree age, some of which are approaching 100 years, while unfavorable weather could have affected fungal establishment. For example, excess rainfall at the beginning of the study (March 2020 = 241 mm and April 2020 = 370 mm) could have washed out the applications, while a following long dry period between June to September in 2020 with less than 10 days of rain during the four months, without irrigation, may have restricted penetration of the drench to lower depths. Abiotic factors are well known to influence the colonization of biocontrol agents in soil and roots (Timper, 2011). In a similar study on mature coffee trees in Brazil, Zinger et al. (2021), evaluated the efficacy of *T. harzianum* against RKN. Unlike our finding, a significant reduction in *M. incognita* was observed in roots after just two months of the application, although no record of fungal colonization in roots was provided. On the other hand, Arita et al. (2020) reported a poor efficacy of *P. lilacinum* and *T. harzianum* to suppress *M. paranaensis* on Arabica coffee in Brazil. The differences in these findings might derive from the different application dosage, methods, RKN species and tested cultivars. Moreover, the use of organic amendments and thus the soil condition can also affect biocontrol colonization, which are often recommended to enhance biocontrol efficacy (Garbeva et al., 2004; Chilosi et al., 2020). In the current study, assessment of the various nematode based indices showed that the soils in which the trials were situated were all generally depleted and stressed, and consequently not ideal for biocontrol establishment. For example, low soil microbial diversity in coffee fields were associated negatively with soil functions and reduced coffee production (Zhao et al., 2018). Application of organic amendments may therefore help in enhancing the efficacy and speed of activity of the biocontrol agents.

The biocontrol agents, *T. asperellum* and *P. lilacinum*, are both common soil inhabitants and suppress RKN in a similar manner, which is mainly through the parasitism of nematode eggs and J2, although *Trichoderma* spp. also produces metabolites, which are nematicidal (Sharon et al., 2001). And yet, in our study, *P. lilacinum* reduced the RKN root population densities more rapidly than *T. asperellum* but then disappeared from soil and roots faster, once the application was stopped. The persistence of biocontrol agents in soil or roots mainly depends on how well they can multiply, where various external factors affect their ability to proliferate, such as edaphic factors, the microbial community structure, and the abundance of the host organisms (Abd-Elgawad and Askary, 2020). It is possible that *P. lilacinum* failed to adapt in the soil and root habitats and thus could not multiply efficiently in the fields of our study. It was not clear from our study as to which factors specifically prevented the longer-term establishment of *P. lilacinum* but for it to proliferate it would likely demand a higher frequency application in order to maintain a similar control efficacy of RKN that *T. asperellum* showed.

The nematode community assay in our study revealed different responses of the nematode fauna to the application of *T. asperellum* and *P. lilacinum.* As both products are fungal based, an increase of fungivores for both treatments was anticipated; however, an increase in fungivores was only obvious for *P. lilacinum.* Furthermore, of the two dominant fungivores, *Aphelenchoides* and *Aphelenchus*, only *Aphelenchus* was responsive to the *P. lilacinum* application, which may imply that *P. lilacinum* is a preferable food source to some genera than others. The increase in fungivores by the *P. lilacinum* application is not necessarily an ideal change to the soil health conditions, however, as the decomposition channel shifts from bacterial to fungal, which results in a slower decomposition process. Nonetheless, it would be important to understand the mechanism behind this phenomenon towards optimizing *P. lilacinum* application. An increased abundance of omnivores, dominated by *Dorylaimid*, and an improvement in maturity indices (i.e. maturity index, maturity index 2-5 and sigma maturity index), Structure index and Shannon index following *T. asperellum* application indicated less stressed, less disturbed and more stable environments with higher diversity (Ferris and Bongers, 2009). The enhanced diversity richness with *T. asperellum* applications also reflects previous studies (e.g. Alexander and Phin, 2014). Despite the more rapid suppression of RKN in roots by *P. lilacinum*, the nematode community assay revealed that *T. asperellum* had more positive impacts on soil health conditions, as demonstrated through the various indices.

The PCA was useful in highlighting the difference in nematode assemblages between the treatments as well as a temporal effect on the genera associated with the treatments. A few observations in the PCA supported the observation above, such as the shift of *Aphelenchus* spp. early in the study from a weak association across treatments to a strong association with the *P. lilacinum* treatment and the strong association of *Meloidogyne* in the control later in the study. On the other hand, the PCA revealed perspectives that were not clear from the generalized linear mixed models. For example, the genus *Mesorhabditis*, which is a c-p 1 group nematode, was initially associated with *T. asperellum* but later showed a strong association with *P. lilacinum.* Nematodes in c-p 1 group are indicative of food-enriched conditions (Bongers and Bongers, 1998), and that the application of *P. lilacinum* may enrich the environmental quality. Furthermore, *Rotylenchulus* showed a strong association with *P. lilacinum* later in the study, which was not confirmed using generalized linear mixed models. The analysis of the nematode community at the genus level, also highlighted the distinguished assemblage associated with the *T. asperellum* treatment, which showed less association with the most abundant nematode genus of this study. The evaluation of the impact of treatments at the genus, or even at species level, can be more insightful to better understand the soil ecosystem as opposed to analysis based on feeding type (Brussaard et al., 2004). The analysis of the nematode community data at multiple levels therefore facilitates an integrated understanding of the soil health conditions and should be considered in similar studies.

Since being proposed by Ferris (2010), the functional metabolic footprint is gaining increasing recognition for the description of food web form and function, and to evaluate the impact of land use (Krashevska et al., 2019), farming systems (Briar et al., 2011; Ilieva-Makulec et al., 2016) and management options (Gupta et al., 2019) on food web conditions. In our study, the analysis was not sensitive enough to capture any differences between treatments. All three treatments fell into the quadrant that indicates stressed conditions, while the temporal factor failed to influence the food web conditions over the two-year period. It is possible that this is due to the high abundance of fungivores, as well as c-p 2 nematodes across our coffee fields. For example, the two most abundant nematode families, Cephalobidae and Aphelenchoididae, are c-p 2 group bacterial scavengers and fungal feeders (Bongers and Bongers, 1998). These general opportunists can quickly adapt to and are associated with stressed conditions (Ferris et al., 2001). The high ratio of fungivores to bacterivores also indicated a fungal based slower decomposition channel rather than bacterial. This thereby reflects depleted, poor quality nutrient availability, resulting in a low Enrichment index value (Ferris et al., 2001). An increased abundance of c-p 3 and c-p 4 group nematodes was observed, however, after fungal colonization was recorded in roots for *T. asperellum.* This indicates that the shift in the food web condition following application of such biocontrol agents is gradual and requires longer-term monitoring to better appreciate the influence of biocontrol applications, especially when dealing with stressed, degraded soils.

In respect to plant-parasitic nematodes, the most commonly occurring and dominant genus was *Meloidogyne*, which was identified solely as *M. hapla.* No other *Meloidogyne* species was detected from any of the coffee fields in the study. *Meloidogyne hapla* has been detected in Kenya on other vegetable crops (Karuri et al., 2017; Chitambo et al., 2018) and has also been identified from neighboring coffee estates (Saikai, personal observation). Among the *Meloidogyne* species reported on coffee globally, *M. exigua, M. incognita*, and *M. paranaensis* are considered the most important (Villain et al., 2018); *M. africana* and *M. decalineata* were reported as important from a study in Tanzania (Bridge, 1984). All the fields in our current study were heavily infested with *M. hapla* with significant root damage observed, unlike the reports of Villain et al. (2013). It has been recorded from coffee elsewhere in the region, such as Democratic Republic of the Congo (Villain et al., 2018) and Tanzania (Bridge, 1984; dos Santos et al., 2019), with some occasional reports from higher altitudes in Latin America (Villain et al., 2013) but has generally been considered to be of limited economic importance on coffee (Lordello, 1982; Bridge, 1984). It is, however, reported to be of significant importance on coffee in Hawaii (Handoo et al., 2005). The current study indicates that *M. hapla* is likely of significant importance on coffee, at least in Kenya, although no studies have so far been conducted to determine this. During our study, RKN soil densities were not necessarily reflective of the actual disease pressure observed on the roots, with which there was a poor correlation between the RKN soil densities and RKN root densities. We often recovered low or even zero RKN densities from soil, even when high RKN densities were observed in roots, and *vice versa*. Consequently, this might be associated with weather conditions to some extent, but there is also potential for some improvements in sampling and nematode extraction techniques. This is particularly relevant as the modified Baermann plate method relies heavily on nematode mobility. Nonetheless, based on our study, assessment of the RKN infection in roots was preferable to soil, to better appreciate pest pressure.

Globally, RKN is considered as the most significant nematode threat to the coffee production (Villain et al., 2018) but there is relatively little knowledge on the distribution of species or damage levels in Africa (Coyne et al., 2018). Bridge (1984) estimated a ~20% loss in yield on coffee due to nematode pests, mainly RKN, in Tanzania, while a survey in Ethiopia (Mekete et al., 2008) found only a limited occurrence of RKN on Ethiopian coffee. Some concern regarding RKN damage to coffee in Kenya has been raised (Nzesya et al., 2014) but in general, there is little understanding of the issue across the coffee sector. In our study, the mean total abundance of herbivores, including the root hair feeders, was just 7%, whereas Nzesya et al. (2014) reported a 64% total abundance of herbivores in their samples. Despite some extensive root galling damage observed in our fields, recovered nematodes from samples were at times relatively low. In general, poorly managed coffee farms tend to be more affected by RKN, compared to well-managed commercial farms (Nzesya et al., 2014).

For practical usage of these biocontrol agents by farmers in the region, the financial and economic viability needs to be established. The frequency of application in our study was conducted to initially establish the fungal antagonists in the soil through a high frequency of application and then to maintain them. More in-depth assessment is required to determine effective and economical establishment rates, and for different soil types. We estimate an approximate product cost of $23.43 USD per tree over the two years of study, plus labor fee. At current 2022 rates, farmers receive approximately $5.00 USD for grade AA dried beans per kilogram. To break even therefore, a yield improvement of approximately 4.7 kg (28 kg fresh cherries) of the best quality dry beans per tree over two years is needed. However, the additional merits of using these biocontrol products would be useful to assess, such as an impact on other pests and diseases and long-term soil health improvement that would improve nutrient use efficiency, for example, due to improved root development and feeder root mass. During sampling of roots towards the latter part of the study, more prolific feeder root growth was visually observed in the biocontrol treatment plots than the control, indicating their influence on root recovery and development. This proved difficult to validate empirically but should be looked at in future studies. Application regimes could also be developed to optimize the application timing, such as targeted application to coincide with the period of root flush, to reduce the frequency. We were unable to measure yield in the current study, as the pruning program/cycle rendered yield data of little value. Instead, yield needs to be monitored over a longer time period, such as five years and a mean per year calculated, to negate the influence of the pruning program. Considering the observed damage caused by RKN alone, it was apparent that root efficiency was significantly compromised and not functioning effectively to access nutrients or water. Neither replacing existing coffee trees with new seedlings nor applying synthetic chemical pesticides are attractive strategies. However, our study has demonstrated that biocontrol agents provide an alternative option for managing nematode diseases on mature and established coffee farms.

## 5 Conclusions

Reversing or arresting even, long-term nematode damage inflicted on the root systems of perennial crops is not readily addressed, nor easily implemented. The decades-long manifestation of nematode pests, such as RKN on mature coffee plantations, has major implications to productivity and the economic viability of aging plantations. Replacing these plantations with new plants has huge financial implications, while the blanket treatment of the soil with synthetic pesticides has major environmental and cost considerations. This current study therefore assessed the potential of drenching biocontrol agents on to established, mature coffee fields in Kenya, all of which were affected by nematode infection and damage. Our study has shown that two locally available biocontrol agents, *Trichoderma asperellum* and *Purpureocillium lilacinum*, can effectively suppress RKN infection on mature coffee plants and improve soil health. These biocontrol agents offer a potential sustainable alternative to chemical inputs for coffee farmers in the region. During a two-year period, the drenching application of *T. asperellum* and *P. lilacinum* products showed a significant reduction in the population density of *Meloidogyne hapla* in roots of established Arabica coffee plantations. Additionally, some improvements were observed in the soil food web structure after the application of these biocontrol agents, even over this relatively short period. Our study showed that *P. lilacinum* was more effective than *T. asperellum* in reducing root population densities of *M. hapla*, although it disappeared from the soil and roots faster once the application was stopped. However, despite the more rapid suppression of *M. hapla* by *P. lilacinum*, the nematode community assay revealed that *T. asperellum* had a more positive impact on soil health conditions, as demonstrated through various nematode based indices. Contrary to previous reports in other regions, our current study suggests that *M. hapla* may be significant in coffee cultivation, particularly in Kenya, although no previous studies have investigated its importance in this context.

## Data availability statement

The raw data supporting the conclusions of this article will be made available by the authors, without undue reservation.

## Author contributions

KS designed the experiment as the project lead. KS implemented and managed the field trials and the sample processing in collaboration with CO and ES. The data collection, especially for the nematode community data, was conducted by KS. KS analyzed the data. The manuscript was drafted by KS. CO contributed to the experimental design and assisted the implementation and management of the field trial with KS. CO counted and identified the plant-parasitic nematodes. ES assisted the implementation and management of the field trials and the sample processing. SN supported the implementation and management of the field trials as well as the fungal isolation from soil and roots. RM provided the biocontrol products evaluated in the study. SH supervised the lead author at *icipe* and provided the research facilities. SH contributed to draft the article and approved the version submitted to the journal. DC supervised the lead author at IITA and provided the research facilities. DC contributed to design of the experiment, draft the article and approved the version submitted to the journal. All authors contributed to the article and approved the submitted version.
